# A Culture-Based Strategy Is More Cost Effective Than an Empiric Therapy Strategy in Managing Pediatric *Helicobacter pylori* Infection

**DOI:** 10.3389/fped.2022.860960

**Published:** 2022-05-03

**Authors:** Chi-Wen Hung, Solomon Chih-Chen Chen, Li-Jung Elizabeth Ku, Bor-Shyang Sheu, Yao-Jong Yang

**Affiliations:** ^1^Department of Pediatrics, Kaohsiung Veterans General Hospital, Kaohsiung, Taiwan; ^2^Department of Pediatrics and Institute of Clinical Medicine, National Cheng Kung University Hospital, Medical College, National Cheng Kung University, Tainan, Taiwan; ^3^Department of Pediatrics, Taitung Christian Hospital, Taitung, Taiwan; ^4^Department of Medicine, School of Medicine, Kaohsiung Medical University, Kaohsiung, Taiwan; ^5^Department of Medicine, School of Medicine, Taipei Medical University, Taipei, Taiwan; ^6^Department of Public Health, College of Medicine, National Cheng Kung University, Tainan, Taiwan; ^7^Department of Internal Medicine, National Cheng Kung University Hospital, College of Medicine, National Cheng Kung University, Tainan, Taiwan

**Keywords:** *H. pylori* treatment, child, cost-effectiveness, culture-based, empiric therapy

## Abstract

**Background:**

*Helicobacter pylori* infection is a major cause of peptic ulcers and gastric cancer. This study aimed to compare the eradication rate and essential costs of culture-based and empiric therapy strategies in treating pediatric *H. pylori* infection.

**Methods:**

We retrospectively enrolled patients aged <18 years with a diagnosis of *H. pylori* infection who received esophagogastroduodenoscopy at two medical centers in southern Taiwan from 1998 to 2018. Patients with positive cultures and minimum inhibitory concentration test results were allocated to a culture-based strategy, and those with negative cultures or without culture as an empiric therapy strategy. We collected demographic data and eradication rates, and calculated the total essential costs of treating a hypothetical cohort of 1,000 pediatric patients based on the two strategies.

**Results:**

Ninety-six patients were enrolled, of whom 55 received a culture-based strategy and 41 received an empiric therapy strategy. The eradication rates with the first treatment were 89.1 and 75.6% in the culture-based and empiric therapy strategy, respectively. There were no significant differences in age, sex, and endoscopic diagnosis between the two strategies. For every 10% increase in those receiving a culture-based strategy, the total cost would have been reduced by US$466 in a hypothetical cohort of 1,000 patients. For every 10% increase in successful eradication rate, the total cost was reduced by US$24,058 with a culture-based strategy and by US$20,241 with an empiric therapy strategy.

**Conclusions:**

A culture-based strategy was more cost effective than an empiric therapy strategy in treating pediatric *H. pylori*-infected patients.

## Introduction

*Helicobacter pylori (H. pylori)* infection can cause gastric diseases in both adults and children. The successful eradication of *H. pylori* infection can cure gastritis and prevent the recurrence of ulcerations and long-term complications such as gastric atrophy, intestinal metaplasia, and gastric cancer (1). Although a continuous decrease in the *H. pylori* infection rate has been reported in many areas of the world, including Taiwan, Korea, China, the Middle East, Austria, and Iran, more than half of the global population are still infected with *H. pylori* (2–5). The prevalence of *H. pylori* infection is mainly related to socioeconomic status and living environment (6, 7). *H. pylori* infection generally occurs during childhood and persists throughout life if untreated (8). Therefore, successful early treatment is desirable.

*H. pylori* infection is usually treated with a combination of proton pump inhibitors (PPIs) and antimicrobial agents. Graham et al. reported that the optimal first-line eradication rate needs to be more than 90% to prevent secondary antimicrobial resistance and further unnecessary interventions and cost in per-protocol analysis (9). However, this goal is rarely achieved in pediatric trials under a test-to-treat strategy. One meta-analysis reported eradication rates of *H. pylori* infection according to pediatric consensus guidelines of only 71.7 and 73.6% with triple regimen treatment for 14 days and sequential regimen treatment for 10 days, respectively (10). However, Silva et al. reported a 97.8% eradication rate with susceptibility-based therapy in *H pylori*-infected children in Portugal (11). Furthermore, the joint ESPGHAN/NASPGHAN guidelines in 2017 recommend that eradication treatment in children should be tailored according to antimicrobial susceptibility (6).

Vakil and Ashorn reported that a test-to-treat strategy for children based on serology did not result in cost savings in developed countries with a low prevalence (12). This conclusion is compatible with another study which suggested that the choice of an initial test for *H. pylori* detection should depend on the prevalence of *H. pylori* infection and the diagnostic accuracy (13). However, few studies have focused on the cost-effectiveness of *H. pylori* eradication therapy in children according to test-to-treat or antimicrobial susceptibility-based therapy. Therefore, the aim of this study was to investigate the cost-effectiveness of a culture and antimicrobial susceptibility-based strategy compared to an empiric therapy strategy for children who receiving esophagogastroduodenoscopy in a country with a high antimicrobial resistance rate.

## Materials and Methods

### Patient Inclusion and Exclusion Criteria

We retrospectively enrolled children aged <18 years who received esophagogastroduodenoscopy for upper gastrointestinal disorders and were diagnosed with *H. pylori* infection at two medical centers in southern Taiwan from 1998 to 2018. Only patients who had not previously received *H. pylori* eradication therapy were included. Patients with a drug compliance rate <80% or lost follow-up for investigating the successful eradication or not were excluded. We classified the patients into two groups according to whether they received a culture-based strategy or empiric therapy strategy. This study was approved by the institutional review board of National Cheng Kung University Hospital (IRB No. NCKUH-A-ER-109243) and Kaohsiung Veterans General Hospital (IRB No. VGHKS-20-CT12-04).

### Esophagogastroduodenoscopy Examination and Diagnosis of *H. pylori* Infection

During esophagogastroduodenoscopy examination, six gastric biopsy pieces were taken. One antral tissue sample was used initially for bacterial culture, and one other piece of antral tissue was used for a rapid urease test. Another two antral and two body pieces were used for histology. Once *H. pylori* had been isolated, an antimicrobial susceptibility test was performed using the E-test method (bioMerieux Inc., Craponne, France). A strain was considered to be resistant if the minimum inhibitory concentration (MIC) was ≥0.125 μg/ml of amoxicillin, ≥1 μg/ml of clarithromycin, ≥8 μg/ml of metronidazole, ≥1 μg/ml of tetracycline, and ≥1 μg/ml of levofloxacin (14).

The diagnosis of *H. pylori* was established according to the following criteria: 1) positive culture, or 2) two or more positive histology results, rapid urease test, or ^13^C-urea breath test (^13^C-UBT). The cutoff value of positive ^13^C-UBT was defined as excess ^13^CO2/^12^CO2 ratio more than 4.0‰. The ^13^C-UBT was validated with a favorable sensitivity and specificity (>95%) in the detection of *H. pylori* infection in children aged >6 years (15). A second ^13^C-UBT was arranged 4–6 weeks after finishing eradication therapy or stopping proton pump inhibitor (PPI) to confirm successful eradication. Demographic data including sex, age, and esophagogastroduodenoscopy findings were obtained retrospectively from medical records.

### Treatment and Duration of *H. pylori* Eradication

All of the patients who received the empiric therapy strategy were treated with triple therapy containing amoxicillin (50 mg/kg/day, max. 1,000 mg bid), clarithromycin (15 mg/kg/day, max. 500 mg bid), and PPIs (esomeprazole 2 mg/kg/day, max. 40 mg bid) for 7 to 14 days as the first-line therapy. If the initial treatment failed, the patients received quadruple therapy for 7 to 14 days containing bismuth subcitrate (8 mg/kg/day, max. 240 mg bid), metronidazole (20 mg/kg/day, max. 500 mg bid), amoxicillin or tetracycline (250–500 mg qid, max. 500 mg qid) for those older than 8 years of age, and PPIs (esomeprazole 2 mg/kg/day, max. 40 mg bid) were used as the second-line therapy. However, three children who failed triple therapy received esophagogastroduodenoscopy again during which tissue cultures were obtained and antimicrobial susceptibility tests were performed, and then the treatment regimen was chosen according to the susceptibility test.

In the culture-based regimen group, treatment with amoxicillin, clarithromycin and PPIs for 7 to 14 days was chosen as the first-line therapy if the antimicrobial susceptibility test showed susceptibility to both amoxicillin and clarithromycin. Treatment with amoxicillin, metronidazole and PPIs was chosen if *H. pylori* was resistant to clarithromycin but susceptible to metronidazole and amoxicillin. If both clarithromycin and metronidazole were resistant, amoxicillin, levofloxacin (10 mg/kg/day, max. 500 mg qd for old child) and PPIs were used. If the susceptibility test showed resistance to amoxicillin, clarithromycin, and metronidazole, quadruple therapy was used as the first choice. If the treatment failed, we chose other susceptible antibiotics based on previous antimicrobial susceptibility as the rescue therapy.

The patients in whom eradication failed after the rescue therapy received a second esophagogastroduodenoscopy, and tissue was obtained for culture and susceptibility tests. As this only involved a small number of patients, we did not take the subsequent costs into account.

### Cost Effectiveness Analysis

All direct costs from diagnosis to successful eradication were considered in this study, including outpatient department visits, esophagogastroduodenoscopy examinations, stomach tissue histology, rapid urease tests, stomach tissue cultures, minimum inhibitory concentration (MIC) tests, medications for eradication, and ^13^C-UBTs. Indirect costs such as traveling to receive health care and loss of work time for the caregiver were also calculated, with the minimum cost including traffic fee, parking fee, and 4 h minimum wage in Taiwan. All of the direct costs were calculated according to National Health Insurance program in Taiwan Supplemental Table 1. We used a Microsoft Excel-based model to calculate the total essential costs and sensitivity analysis. SPSS Statistics version 21 (IBM SPSS Statistics for Windows, Version 21.0. Armonk, NY, USA) was used for statistical analysis.

### Statistical Analysis

**Figure 1:**
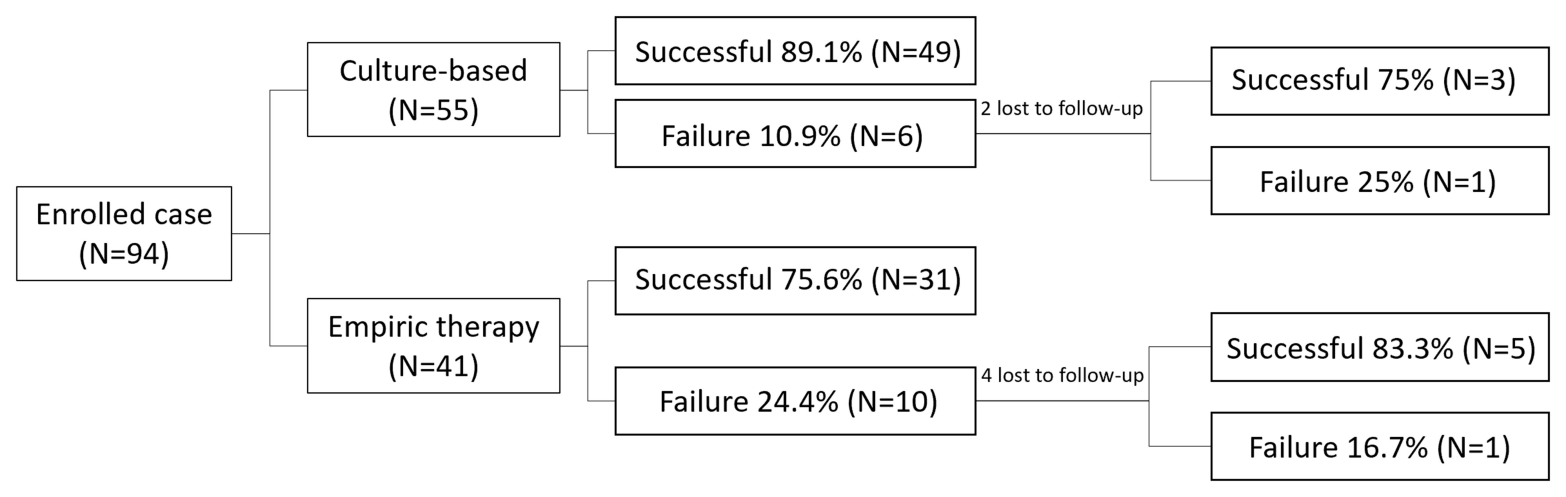
The eradication rates and number of cases in the two groups.

Comparisons of both strategies and cost-effectiveness analysis were conducted. The statistics we used in this study were *t-test* for age, chi-squared test for sex, and endoscopic diagnoses. Both are parametric tests. A decision analytical tree was used to calculate the overall cost of each strategy (Figure 1) and to describe the cost per unit health improvement with each treatment strategy. In this study, the eradication rate of each intervention after two courses of treatment was considered to be the target unit. A *p value* <0.05 defined as a statistic significance.

## Results

### Demographic Data and Eradication Rate in the Two Groups

A total of 94 dyspeptic children were included in this study, of whom 53 received stomach tissue cultures and MIC tests (the culture-based group) and the other 41 did not receive cultures or were culture-negative (the empiric therapy group). There were no significant differences in sex (*p* = 0.26), age (13.5, range 6.2–18 vs. 14.2, range 3.7–18 years, *p* = 0.75), and esophagogastroduodenoscopy diagnosis (duodenal ulcer: 46 vs. 56%; gastric ulcer: 15 vs. 5%; gastritis: 40 vs. 39%, *p* = 0.24) between the culture-based group and the empiric therapy group. The eradication rates for the first treatment were 89.1% (49/55) and 75.6% (31/41) in the culture-based and empiric therapy strategy groups, respectively (Figure 1).

In the culture-based group, two of the six patients who failed treatment were lost to follow-up at the outpatient department. The other four received a second course of eradication treatment according to a previous susceptible test. Three of the four patients achieved successful eradiation after the second course of treatment (eradication rate: 75% [3/4]). In the empiric therapy group, four of the 10 patients who failed treatment were lost to follow-up, and the other six received a second course of eradication treatment with quadruple therapy. Five of the six patients achieved successful eradicated (eradication rate: 83.3% [5/6]).

### Antimicrobial Susceptibility of *H. pylori* Isolates

The overall resistant rates to amoxicillin, clarithromycin, metronidazole, and levofloxacin of 55 *H. pylori* isolates from children without previous eradication therapy were 1.8, 20, 23.6, and 4.4%, respectively. Among them, only 45 of 55 isolates were tested for levofloxacin and tetracycline. None were resistant to tetracycline. The dual resistant (clarithromycin + metronidazole) and triple resistant (Amoxicillin + clarithromycin + metronidazole) rates of isolates were 10.9 and 1.8%, respectively. The changes of antimicrobial resistant rates of *H. pylori* isolates in the past two decades had have been shown in previous study (14).

### Culture-Based Strategy Had a Favorable Cost Per Eradication

**Figure 2:**
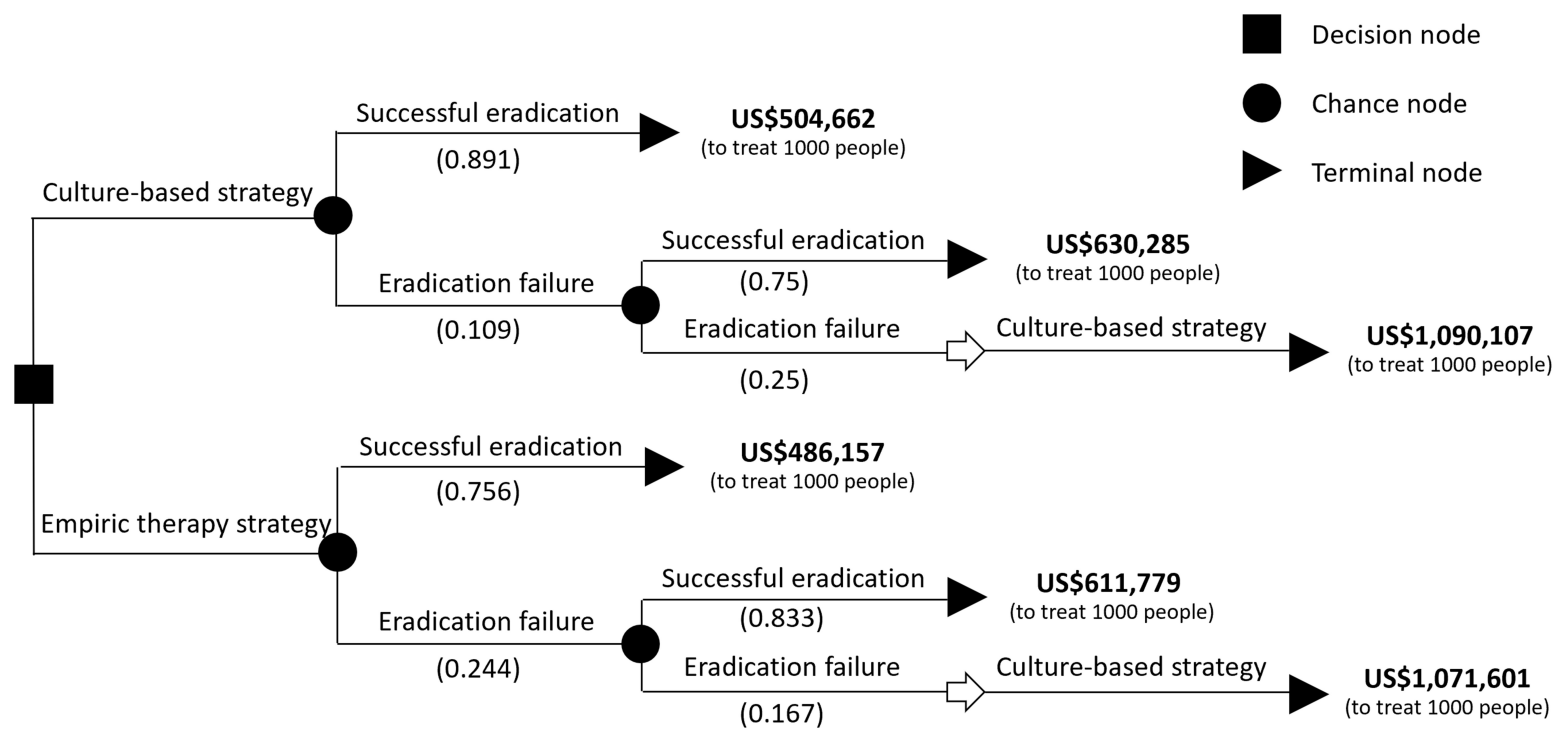
The decision tree and eradication rate of the two eradication strategies. The probability of each outcome is noted beside the branches. The cost behind the terminal node was calculated in treating 1,000 infected children. The total cost of treating 1,000 infected children with a culture-based strategy was calculated as 504,662 x 0.891 + (630,285 x 0.75 x 0.109 + 1,090,107 x 0.25 x 0.109).

The *H. pylori* eradication decision tree (Figure 2) maps the components of the total costs of each strategy to treat 1,000 patients, as a result of the financial burden of both successful eradication and failure after two courses of treatment. The total cost per strategy for 1,000 patients and associated costs per eradication case are summarized in Table 1. The culture-based strategy had a more favorable cost per eradication than the empiric therapy strategy.

**Table 1:** The eradication rates, total cost to treat 1,000 patients, and cost per eradication with both eradication strategies.

**Strategy**	**Eradication rate^†^ (%)**	**Total cost (US$)**	**Cost per eradication case**	**Incremental cost-effectiveness ratio**
Test-to-treat	95.9	535,545	558.4	-
Culture-based	97.2	530,885	546.2	dominant

† *Total eradication rate after second-line treatment or after two treatments*.

When considering the hypothetical cohort of 1,000 patients, the culture-based strategy would save US$4,660 and result in 135 more cases achieving successful eradication compared to the empiric therapy strategy, and the incremental cost-effectiveness ratio (ICER) was dominant. In other words, the cost-effectiveness was unequivocal and the culture-based strategy achieved better outcomes at a lower cost.


$$\begin{array}{r} ICER=\frac{Costs_{culture-based}-Costs_{test-to-treat}}{{eradiation\;cases}_{culture-based}-{eradication\;cases}_{test-to-treat}} \end{array}$$

### The Relationship Between Percentage of Those Receiving a Culture-Based Strategy and Cost

**Figure 3:**
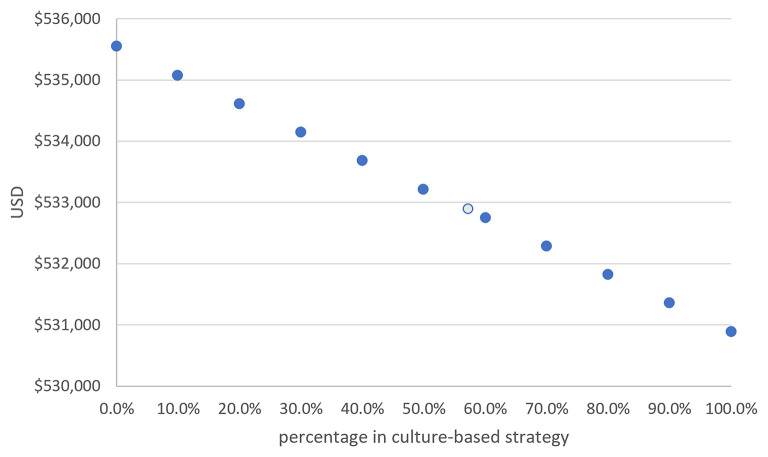
The total cost of treating 1,000 *H. pylori*-infected children was negatively correlated to the percentage of those receiving a culture-based strategy. For every 10% increase in those receiving a culture-based strategy, the total cost was reduced by US$466 in 1,000 patients (The light-blue circle indicates our series).

Overall, 42.7% (*n* = 41) of the patients received an empiric therapy strategy and 57.3% (*n* = 55) received a culture-based strategy. Figure 3 shows that a 10% increase in those receiving a culture-based strategy would save US$466 when treating 1,000 *H. pylori*-infected patients. Likewise, a 20% increase in those receiving a culture-based strategy would save US$932 and 30% would save US$1,398. The total costs were inversely correlated to the percentage of those receiving a culture-based strategy. That is, a higher percentage of patients receiving a culture-based strategy for pediatric *H. pylori* infection would result in the treatment being more cost effective.

### The Relationship Between Eradication Rate of the Two Strategies and the Cost

**Figure 4:**
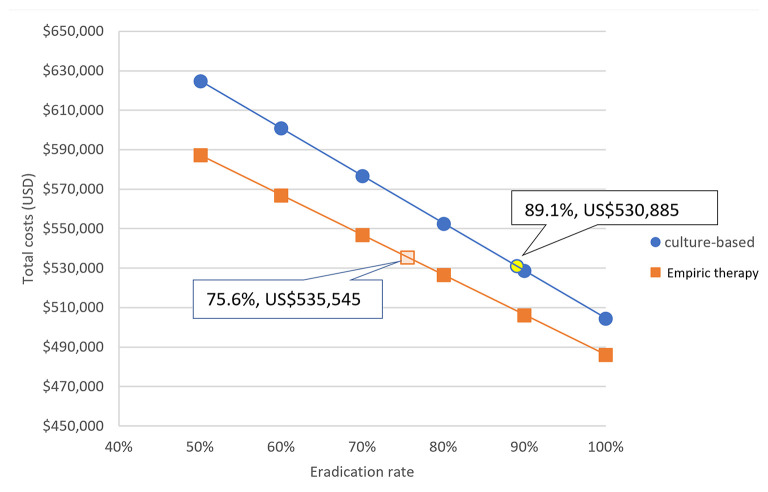
The impact of eradication rate on the costs of the two eradication strategies. For every 10% increase in successful eradication rate, the total cost was reduced by US$24,058 with a culture-based strategy and by US$20,241 with an empiric therapy strategy (Both indicated successful eradication rates and costs are our series).

Because the eradication rate with a culture-based strategy was superior to that of an empiric therapy strategy, we investigated the impact of eradication rate on the costs of the two eradication strategies. Figure 4 shows that the total cost of treating 1,000 *H. pylori*-infected children was negatively correlated to the successful eradication rate with both strategies.

## Discussion

This is the first cost-effectiveness study of treatment for pediatric *H. pylori* infection comparing culture-based and empiric therapy strategies. The results showed that a culture-based strategy was more cost effective in eradicating pediatric *H. pylori* infection than an empiric therapy strategy. Therefore, a culture-based method was beneficial in both increasing the eradication rate and saving costs in the management of pediatric *H. pylori* infection.

Previous studies have reported that treating *H. pylori* infection according to culture and susceptibility tests resulted in eradication rates ranging from 89.9 to 97.8% in pediatric patients (11, 16). However, other studies have reported eradication rates with a Non-culture-based strategy with triple therapy and sequential therapy of 75.5 and 80.4%, respectively (17, 18). The lower eradication rates with both triple and sequential therapies are thought to be due to the increase in antibiotic-resistant rates (17). As shown in the current and previous studies (14), a high resistant rate to clarithromycin and metronidazole in pediatric *H. pylori* isolates implies that the common used triple therapy will result in eradication failure in Taiwan. Therefore, to achieve a 90% eradication rate, culture and susceptibility tests are recommended when treating children with *H. pylori* infection (6, 11, 16, 19), especially in areas with a high prevalence of resistant *H. pylori* strains (20). In addition, it is difficult to perform a second esophagogastroduodenoscopy in children if the initial treatment was not successful (21). These reasons strongly suggest that tissue cultures should be done during the first esophagogastroduodenoscopy to allow for appropriate treatment. There are some studies investigating the cost-effectiveness for *H. pylori* eradication therapy between culture/susceptibility and empirical therapies (7- or 10-day triple therapy) in adults (22–24). The conclusions are controversial. The inconsistent results could due to choose of different first-line therapy (25). However, to the best of our knowledge, no previous study has compared the cost-effectiveness between culture-based and empiric therapy strategies for pediatric *H. pylori* treatment.

In this study, the culture-based strategy showed a cost saving of US$4,660 compared to an empiric therapy strategy when treating a hypothetical cohort of 1,000 Taiwanese pediatric patients. This does not seem like a significant cost reduction with a culture-based strategy over an empiric therapy strategy. However, the medical costs in Taiwan are far cheaper when compared with Western countries. For example, the cost of an esophagogastroduodenoscopy in Taiwan is US$54 (currency exchange rate for the New Taiwan dollar (NT$) to the US$ as of December 2020: NT$28.1 = US$1) according to the Taiwan Health Insurance Agency. In contrast, the average cost of an esophagogastroduodenoscopy is US$1,557 (ranging from $1,250 to $4,800) in the United States based on Medicare data (https://www.medicare.gov/). Similarly, outpatient visits, venous anesthesia, and other direct and indirect costs are higher in Western countries. This means that the cost savings will be more substantial in Western countries.

In addition, the 2017 ESPGHAN/NASPGHAN guidelines recommend that clinicians should avoid clarithromycin as the first-line therapy in areas with a high prevalence of antimicrobial resistance (6). In Taiwan, Lu et al. reported increased resistance rates to clarithromycin and metronidazole for pediatric *H. pylori* in the past decade (14). This mean that eradication failure rate may increase if using a nonculture-based strategy as the first-line treatment. Therefore, the difference in effectiveness between the two treatment strategies will be more obvious. Our results also confirm the ESPGHAN/NASPGHAN guidelines in that a culture-based strategy should be used as the first-line treatment for pediatric *H. pylori* infection to improve the eradication rate (6). Taking together, appropriate treatment should be based on antimicrobial susceptibility to improve eradication rate and cost savings.

A disadvantage for choosing culture/susceptibility method is time wasting. It usually takes about 7–10 days to obtain the final report. We feel this waiting time is valuable for the overall benefit on patients who need to receive *H. pylori* eradiation therapy. In addition, because this study focuses on the comparison of monetary cost, time-cost is not considered. This is a limitation in this study.

Poor compliance due to the adverse effects of antibiotics is also an important factor for eradication failure (16, 26). The eradication rate with a culture-based strategy in this study was only 89.1%, less than the recommended goal (90%) in the ESPGHAN/NASPGHAN guidelines (6). Kotilea et al. reported that the eradication rate of *H. pylori* infection was directly influenced by adherence to therapy in children (80 vs. 36.6%) (16). Therefore, choosing a simple drug combination with fewer adverse effects may achieve a higher eradication rate and cost savings.

There are some limitations to this study. First, this study was a Non-randomized retrospective design, and the two strategies were allocated according to a personal treatment strategy. Second, our data were collected over two decades, and *H. pylori* drug susceptibility has changed subtly over time (14). This may have led to overestimation of the eradication rate with the empiric therapy strategy. Third, we did not perform a cost-utility or cost benefit analysis, by recording differences in long-term health and financial benefits of pediatric *H pylori* eradication.

In conclusion, a culture-based strategy with antimicrobial susceptibility tests for the management of pediatric *H. pylori* infection can achieve a better eradication rate and superior cost-effectiveness than an empiric therapy strategy in areas with a high prevalence of resistant *H. pylori* strains.

## Data Availability Statement

The original contributions presented in the study are included in the article/Supplementary Material, further inquiries can be directed to the corresponding author.

## Ethics Statement

The studies involving human participants were reviewed and approved by National Cheng Kung University Hospital (IRB No. NCKUH-A-ER-109243) and Kaohsiung Veterans General Hospital (IRB No. VGHKS-20-CT12-04). Written informed consent from the participants' legal guardian/next of kin was not required to participate in this study in accordance with the national legislation and the institutional requirements.

## Author Contributions

C-WH: involved in the study design, study conduction, interpretation of data, and in drafting the manuscript. S-CC: involved in the study design and conduction, interpretation of data, and editing the manuscript. L-JK: involved in the interpretation of data. B-SS: involved in the study discussion and editing. Y-JY: involved in the setting of the study design and conduction, interpretation of data, editing, and final approval of the manuscript. All authors contributed to the article and approved the submitted version.

## Funding

This study was supported by a grant (NCKUH-A-ER-109243) from the National Cheng Kung University Hospital, Tainan, Taiwan, and a grant (VGHKS-20-CT12-04) from Kaohsiung Veterans General Hospital, Kaohsiung, Taiwan.

## Conflict of Interest

The authors declare that the research was conducted in the absence of any commercial or financial relationships that could be construed as a potential conflict of interest.

## Publisher's Note

All claims expressed in this article are solely those of the authors and do not necessarily represent those of their affiliated organizations, or those of the publisher, the editors and the reviewers. Any product that may be evaluated in this article, or claim that may be made by its manufacturer, is not guaranteed or endorsed by the publisher.

## Supplementary Material

Supplementary Table 1 The direct and indirect costs of diagnosis and treatment for children with *H. pylori* infection according to the two eradication strategies in Taiwan.
