# Supplementary material for: A Culture-Based Strategy Is More Cost Effective Than an Empiric Therapy Strategy in Managing Pediatric Helicobacter pylori Infection
Source: Front Pediatr. 2022 May 3;10:860960. doi: 10.3389/fped.2022.860960 (PMC9110685; doi:10.3389/fped.2022.860960)
Supplement: Supplementary Table 1 — The direct and indirect costs of diagnosis and treatment for children with H. pylori infection according to the two eradication strategies in Taiwan. [file Table_1.pdf]

Supplemental Table 1 The direct and indirect costs of diagnosis and treatment for children with *H. pylori* infection according to the two eradication strategies in Taiwan.

**Intervention**	**Direct cost (US$†)**
An outpatient visit	29.5
Venous anesthesia	142.5
Esophagogastroduodenoscopy	53.5
Rapid urease test	7.0
Histology	62.0
Culture	7.0
Antimicrobial susceptibility (MICs)	11.5
C^{13} urea breath test	41.5
Drugs (mean)	24.5
	**Indirect cost (US$†)**
Minimum wage for 4 hours in Taiwan	21.5
Minimum two-hour parking fee for an outpatient visit	1.5

†Currency exchange rate for the New Taiwan dollar (NT$) to the US$ as of December 2020: NT$28.1 = US$1.
